# MicroRNA‐125a inhibits tumorigenesis by targeting Smurf1 in colorectal carcinoma

**DOI:** 10.1002/2211-5463.12680

**Published:** 2019-06-17

**Authors:** Dongbin Li, Xiangmei Xu, Jihao Miao, Jianhui Cai

**Affiliations:** ^1^ Department of Gastrointestinal Surgery The Second Hospital of Hebei Medical University Shijiazhuang China; ^2^ Department of Cardiology The No. 1 Hospital of Shijiazhuang China; ^3^ Department of General Surgery The Fourth Hospital of Hebei Medical University Shijiazhuang China; ^4^ Hebei Medical University Shijiazhuang China; ^5^ The Forth Department of General Surgery Hebei General Hospital Shijiazhuang China

**Keywords:** colorectal carcinoma, miR‐125a, Smurf1, tumorigenesis

## Abstract

Aberrant expression of microRNAs (miRNAs) may contribute to the initiation and development of multiple types of human cancer. Several miRNAs have been found to be strongly correlated with the diagnosis, progression, and prognosis of colorectal carcinoma (CRC), but the role of miR‐125a in CRC remains unclear. In the present study, the function of miR‐125a on the expression of Smad ubiquitin regulatory factor 1 (Smurf1) was investigated *in vitro* and *in vivo*. We verified that Smurf1 is a downstream target gene of miR‐125a and is involved in miR‐125a‐mediated regulation of CT26 cell (colon cancer cell) proliferation and migration. Overexpression of miR‐125a suppresses CT26 cell growth by inhibiting cell proliferation. Additionally, wound healing assays were performed to show that overexpression of miR‐125a significantly reduced CT26 cell migration, which was reversed by overexpression of Smurf1. *In vivo*, miR‐125a overexpression downregulated the expression of Ki67 and Smurf1, thus leading to a marked reduction in tumor growth. These results revealed that miR‐125a plays a critical role in CRC by directly targeting Smurf1, a finding that may facilitate the development of improved diagnostic and therapeutic techniques for CRC.

**Abbreviations**
CRC: colorectal carcinoma
DMEM: Dulbecco's modified Eagle's medium
miRNA: microRNA
Smurf1: Smad ubiquitin regulatory factor 1
TGFβ: transforming growth factor beta

Colorectal carcinoma (CRC) is the third most common type of cancer worldwide, causing > 500 000 mortalities annually 1, 2. Several risk factors have been shown to be implicated in the progression of CRC, including lifestyle, environmental factors, and genetic polymorphisms 3. CRC follows the sequential process from adenoma to carcinoma, with significant morbidity and mortality. It has been reported, however, that the introduction of CRC screening programs has reduced the incidence of CRC 4, 5, 6. However, the optimal screening strategy has not yet been identified. It is therefore critical to develop new screening tools as potent biomarkers for CRC.

Recently, certain studies have indicated that microRNAs (miRNAs) are strongly associated with the diagnosis, progression, and prognosis of CRC 7, 8. miRNAs, a class of short noncoding RNAs, serve as important regulators of human gene expression 9. There are no < 1500 human miRNAs in the miRBase database, playing a critical role in post‐transcriptional modification of gene expression via targeting the 3′UTR of specific mRNA 10, thereby affecting certain cellular processes in embryonic development and disease conditions 11, 12, 13. miR‐143 and miR‐145 were the first miRNAs found to be downregulated in precancerous and neoplastic colorectal tissue 14. miR‐143 could act as inhibitor in CRC cell proliferation by targeting K‐ras 15. Schepeler *et al.* 16 showed that the overexpression of miR‐145 could inhibit the three different cells lines of CRC, indicating that it may function as a tumor suppressor. Furthermore, certain miRNAs, such as miR‐192 and miR‐215, have been found to play an important role in carcinogenesis by modulating proliferation, invasion, tube formation, the cell cycle, and angiogenesis 17, 18.

In humans, there are two existing isoforms of miR‐125: miR‐125a and miR‐125b. Several studies have shown that miR‐125b was expressed in prostate, breast, and pancreatic cancers 19, 20. miR‐125b appears to directly bind to the 3′UTR of the P53 gene and contributes to the development and progression of human cancers 21. Nishida *et al.* 22 reported that miR‐125b served as a key prognostic biomarker in colorectal cancer and revealed the underlying mechanism between miR‐125b and CRC.

Smad ubiquitin regulatory factor 1 (Smurf1), belonging to the neural precursor cell expressed developmentally downregulated 4 subfamily of homologous to the E6‐AP C terminus type E3 ubiquitin protein ligases, is encoded by a distinct gene located at chromosome 7, which was originally identified as a negative regulator of the bone morphogenic protein/transforming growth factor beta (TGFβ) signaling pathway in mammals. It has been shown to play a crucial role in embryogenesis and adult tissue homeostasis and has a very high homology and amino acid identity (95%), both murine and human. This suggests that mouse models can be used to appropriately investigate the biological roles of smurfs in humans 23. In the present study, Smurf1 was also found to be the potential target gene of miR‐125a in CRC.

However, to date, the role of miR‐125a in CRC tumorigenesis remains poorly understood and the potential target of miR‐125a in CRC has not yet been fully characterized. The aim of this study was to investigate the effect of miR‐125a on CRC and explore the underlying downstream targets involved.

## Materials and methods

### Cell culture

Mouse colon cancer CT26 and human colon cancer SW620 cells were purchased from the American Type Culture Collection (Manassas, VA, USA) and maintained in Dulbecco's modified Eagle's medium (DMEM) with 10% FBS (Gibco; Thermo Fisher Scientific, Inc., Waltham, MA, USA) under the condition of 5% CO_2_ at 37 °C.

### Bioinformatics analysis

targetscan software (http://www.targetscan.org/) was used to predict the target sites of miR‐125a.

### Western blot analysis

In brief, the total proteins of cells and tissues were extracted using the RIPA buffer (Solarbio, R0010, Shanghai, China) with inhibitor cocktail (Sigma, P8340‐5ML, St. Louis, MO, USA) and the determination of protein concentration by BCA protein assay (SBJ‐1001; Beyotime, SENBEIJIA Bio, NanJing, China). Loading buffer was added to adjust the concentration. After denaturation, add 20 μg to each sample hole. Twelve percent SDS/PAGE was used to separate protein samples, and then, a PVDF membrane was employed to transfer the corresponding proteins, after electrophoresis and transmembrane, sealed for 2 h by skimmed milk. The membrane was incubated with anti‐Smurf1 (PB0937, 1 : 1000; Wuhan Boster Biological Technology, Ltd., Wuhan, China) and anti‐β‐tubulin (2146, 1 : 1000; Cell Signaling Technology, Inc., Danvers, MA, USA) primary antibodies. β‐Tubulin was used as the loading control. Overnight incubation at 4 °C. Washed the PVDF membrane for 3 h, add horseradish peroxidase‐labeled anti‐rabbit antibody (Anti‐Rabbit IgG, HRP‐Linked Antibody 6402‐05, 1 : 5000; Amylet Scientific Inc, Wuhan, China), and incubated at 37 °C for 1–2 h. The bands were visualized using the ECL chemiluminescence colorant (Haigene, M2301, Harbin, China). The grayscale ratio of target protein β‐tubulin was analyzed. It indicates the relative content of the target protein.

### Reverse transcription‐quantitative polymerase chain reaction (RT‐qPCR)

Total RNA was extracted with traditional TRIzol method. GoScript^TM^ Reverse Transcription System (Promega, Madison, WI, USA) was used for synthesis of cDNA. mRNA concentration was assessed by photometric analyses by NanoDrop ND‐1000 UV‐Vis Spectrophotometer (Thermo Scientific, Wilmington, DE, USA).Quantitative RT‐qPCR experiments were performed on a 7300 device (Applied Biosystems, Foster City, CA, USA). At an initial denaturation at 95 °C for 10 min, amplification was performed during 40 cycles of denaturation at 95 °C for 15 s and annealing/extension at 60 °C for 1 min. The primers, control, and quantification method used in qPCR were as those described in reference 24.

### Dual‐luciferase assays

The CT26 cells were seeded into a 24‐well plate and cotransfected with a reporter gene [pMIR‐Smurf1‐wild‐type (WT) or pMIR‐Smurf1‐mutant (Mut)] together with either miR‐125a mimics, control, or miR‐125a inhibitor using Lipofectamine® 2000 (Gibco; Thermo Fisher Scientific, Inc.). The following sequences were used: miR‐125a mimics: 5′‐UCCCUGAGACCCUUUAACCUGUGA‐3′; miR‐125a inhibitor: 5′‐UCACAAGUUAGGGUCUCAGGGA‐3′; miR‐NC: 5′‐UUCUCCGAACGUGUCACGUTT‐3′.

At 24 h post‐transfection, luciferase assays were conducted using the Dual‐Luciferase Assay Kit (Promega Corporation), following the manufacturer's instructions.

### Cell proliferation and wound healing assay

For the cell proliferation assay, cells were plated in a 96‐well plate and a CCK‐8 kit (Dojindo Molecular Technologies, Inc., Kumamoto, Japan) was employed to detect cell proliferation, following the manufacturer's instructions. Absorbance was measured at 450 nm using a microplate reader. For the migration assay, cells (5 × 10^5^) were added to a six‐well plate and incubated for 24 h. A pipette tip was used to scrap the cell monolayer to cause a wound the width of which was photographed 12 h postwounding using a microscope to detect cell migration.

### Mouse xenograft model

CT26 cells treated with miR‐125a agomir or the control were cultured in the fresh DMEM under the normal conditions. Male nude mice (age, 5–6 weeks; weight, 16–19 g; Jackson Laboratory, Bar Harbor, ME, USA) were randomly divided into two groups (*n* = 6 per group): the control (inoculated with control CT26 cells) and the observation group (inoculated with miR‐125a agomir CT26 cells). These nude mice were under the conditions of 22 °C constant temperature, 35–75% humidity, free access to food and water, and 12‐h light/dark cycle. Briefly, cells were collected and suspended in PBS at a concentration of 5 × 10^6 ^cells per ml, and the nude mice were then treated with a subcutaneous injection of cells (5 × 10^5 ^cells, 100 μL). After 6 days, tumor growth was observed every 3 days. The tumor volume (*V*) was calculated using the following formula: *V* = (*L* × *W* ^2^) × 0.5, where *L* and *W* stand for length and width, respectively. Euthanasia was carried out by cervical dislocation after rendering nude mice consciousless with CO_2_, and tumors were dissected and weighed. Subsequently, tumor tissue was preserved in 4% paraformaldehyde for further analysis. The present study was approved by the Ethics Committee of The Second Hospital of Hebei Medical University.

### Immunohistochemical analysis

Tumor tissues from mouse xenograft models were fixed, dehydrated, embedded in paraffin, and sectioned. Tumor tissue slices (4.5 μm) were incubated with anti‐Ki67 (1 : 100; A01K0054; Wuhan Boster Biological Technology, Ltd.) for 2 h at 37 °C. Following washing with PBS, the sections were treated with secondary antibodies (1 : 500; 10600‐P07E‐H; Wuhan Boster Biological Technology, Ltd.) for 1 h at room temperature. Normal colon tissue was used as negative control. Finally, the tumor slices were counterstained with hematoxylin and eosin (H&E) staining and photographed using light microscopy.

### Statistical analysis

The statistical analysis was performed using spss software (SPSS for windows 17.0; SPSS, Inc., Chicago, IL, USA). Data are presented as mean ± SD. Student's *t*‐test was used to compare the mean values between the two groups; one‐way ANOVA was used to compare the mean values among three or more groups, in which the *post hoc* test was Bonferroni. *P* < 0.05 was considered to indicate a statistically significant difference.

## Results

### miR‐125 Regulates Smurf1 expression by targeting the 3′‐UTR of Smurf1

**Figure 1:**
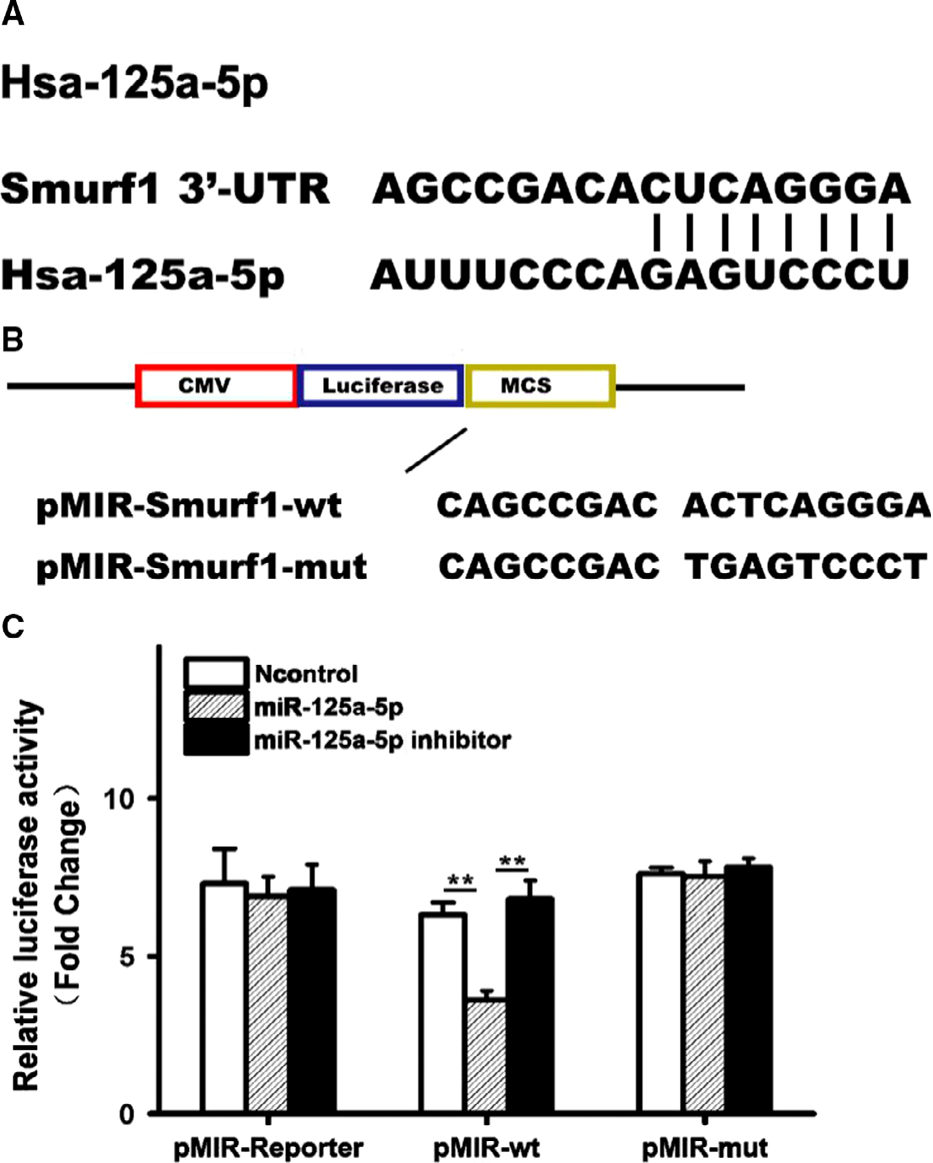
*In vitro* miR‐125a regulates Smurf1 expression by binding to the 3′‐UTR of Smurf1. (A) Schematic representation of the Smurf1 3′‐UTR as a direct target for miR‐125a. (B) WT or Mut miR‐125a target sssss of the Smurf1 3′‐UTR. (C) Luciferase activity of cells cotransfected with the WT or Mut CDKN1A 3′‐UTR reporter genes or negative control miRNA mimics (pMIR‐reporter). Three experiment replicates were performed. Data are presented as mean ± SD. One‐way ANOVA. ^**^ *P* < 0.01.

First, the 3′‐UTR of Smurf1 was screened using the TargetScan (http://www.targetscan.org/) to find a potential miRNA binding site; a putative binding site was identified at the 3′‐UTR of Smurf1 (Fig. 1A).

In order to gain insights into whether miR‐125 successfully targeted the Smurf1 gene, the WT reporter gene was mutated to obtain a reporter vector containing Mut miR‐125 target sequences of Smurf1 (Fig. 1B). Next, CT26 cells were cotransfected with the pMIR‐Smurf1‐WT or pMIR‐Smurf1‐Mut reporter together with either miR‐125a mimics, the control, or miR‐125a inhibitor, and luciferase assays were performed. The transfection of miR‐125a mimics led to a significant reduction in the luciferase activity of the WT 3′UTR reporter gene, compared with the controls, whereas the use of an miR‐125a inhibitor significantly reversed this response (Fig. 1C). However, miR‐125a mimics and the miR‐125a inhibitor failed to affect the luciferase activity of the Mut reporter gene (Fig. 1C).

**Figure 2:**
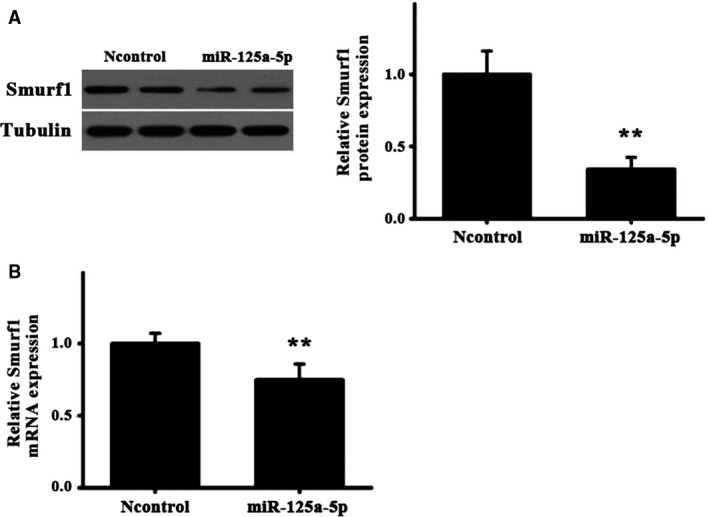
Expression of Smurf1 in cells transfected with miR‐125a. (A) The Smurf1 protein expression and quantification in CT26 cells transfected with miR‐125a or control was determined by western blotting. ^**^ *P* < 0.01. (B) Quantification of Smurf1 mRNA levels was determined by reverse transcription‐quantitative polymerase chain reaction. Data are presented as mean ± SD. Student's *t*‐test. ^**^ *P* < 0.01.

To examine the role of miR‐125a in regulating the expression of Smurf1, we examined the protein and the mRNA expression of Smurf1 using western blot analysis and RT‐qPCR in CT26 cells transfected with miR‐125a mimics or the controls. As expected, the treatment with miR‐125a mimics significantly reduced the mRNA and protein expression of Smurf1, compared with the controls (Fig. 2A,B). These results suggested that miR‐125a may repress the expression of Smurf1 by directly targeting the 3′UTR of Smurf1.

### miR‐125a inhibits cell proliferation and migration in CT26 and SW620 cells

**Figure 3:**
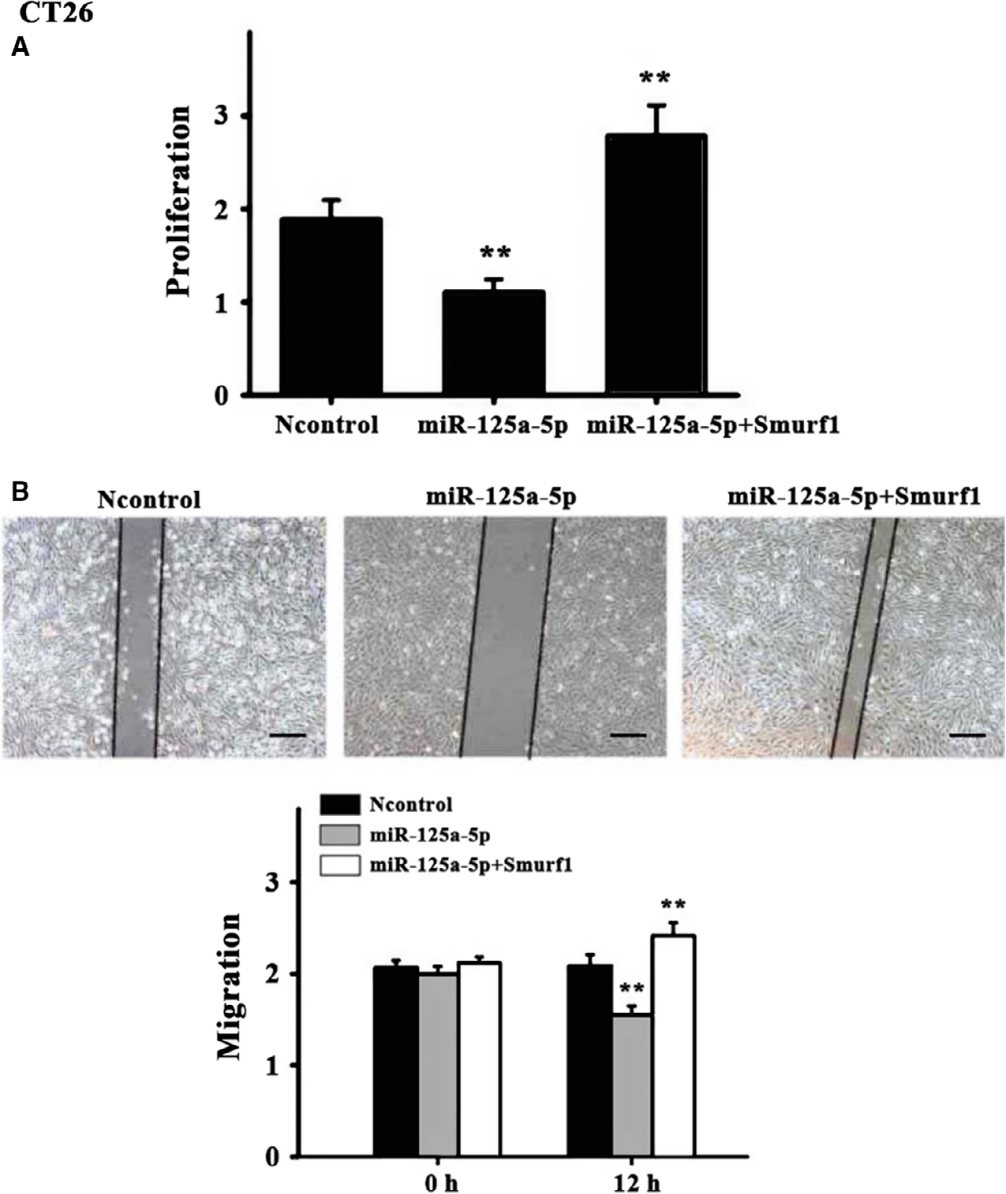
Effects of miR‐125a in CT26 cells. (A) Cell proliferation assays were performed in CT26 cells transfected with Smurf1 and/or miR‐125a‐5p using the CCK‐8 kit. (B) Representative images and quantification of the migration of CT26 cells transfected with miR‐125a, control, or miR‐125a + Smurf1. Scale bar = 25 μm; *N* = 6 per group. Three experiment replicates were performed. Data are presented as mean ± SD. One‐way ANOVA. ^**^ *P* < 0.01.

**Figure 4:**
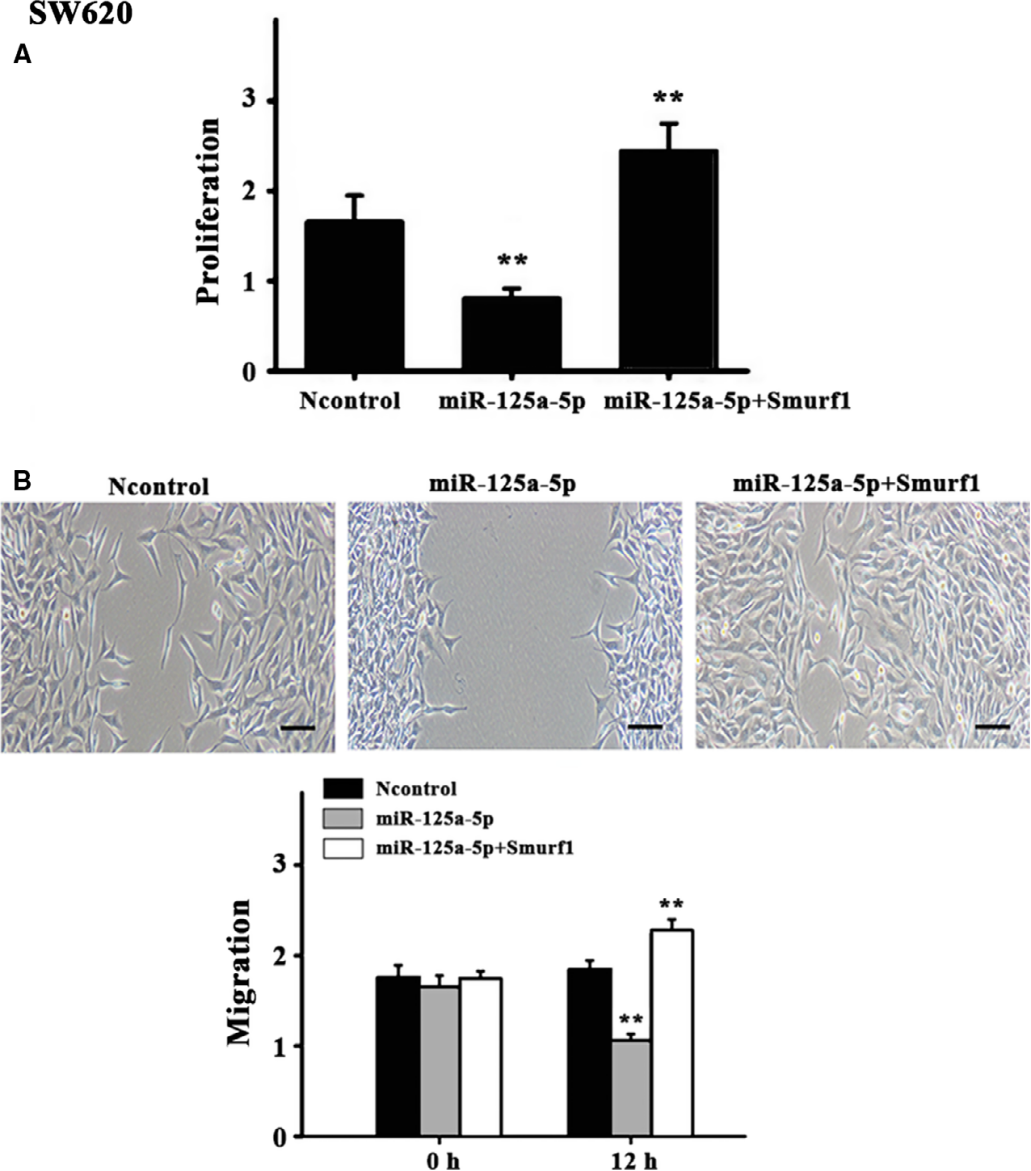
Effects of miR‐125a in SW620 cells. (A) Cell proliferation assays were performed in SW620 cells transfected with Smurf1 and/or miR‐125a‐5p using the CCK‐8 kit. (B) Representative images and quantification of the migration of SW620 cells transfected with miR‐125a, control, or miR‐125a + Smurf1. Scale bar = 25 μm; *N* = 6 per group. Three experiment replicates were performed. Data are presented as mean ± SD. One‐way ANOVA. ^**^ *P* < 0.01.

Considering that miR‐125a negatively influenced Smurf1 expression, it was hypothesized that miR‐125a could inhibit tumor cell proliferation and migration. To assess the impact of miR‐125a on the aspect of cell proliferation, CT26 and human colon cancer cell SW620 cells were transfected with miR‐125a mimics or the control, respectively. Cell proliferation assays with the CCK‐8 kit indicated that the overexpression of miR‐125a significantly inhibited cell proliferation in CT26 and SW620 cells (Figs 3A and 4A). The wound healing assays suggested that the overexpression of miR‐125a significantly decreased the migration of CT26 and SW620 cells, which could be reversed by Smurf1 overexpression (Figs 3B and 4B).

### miR‐125a inhibits tumor growth in nude mice

**Figure 5:**
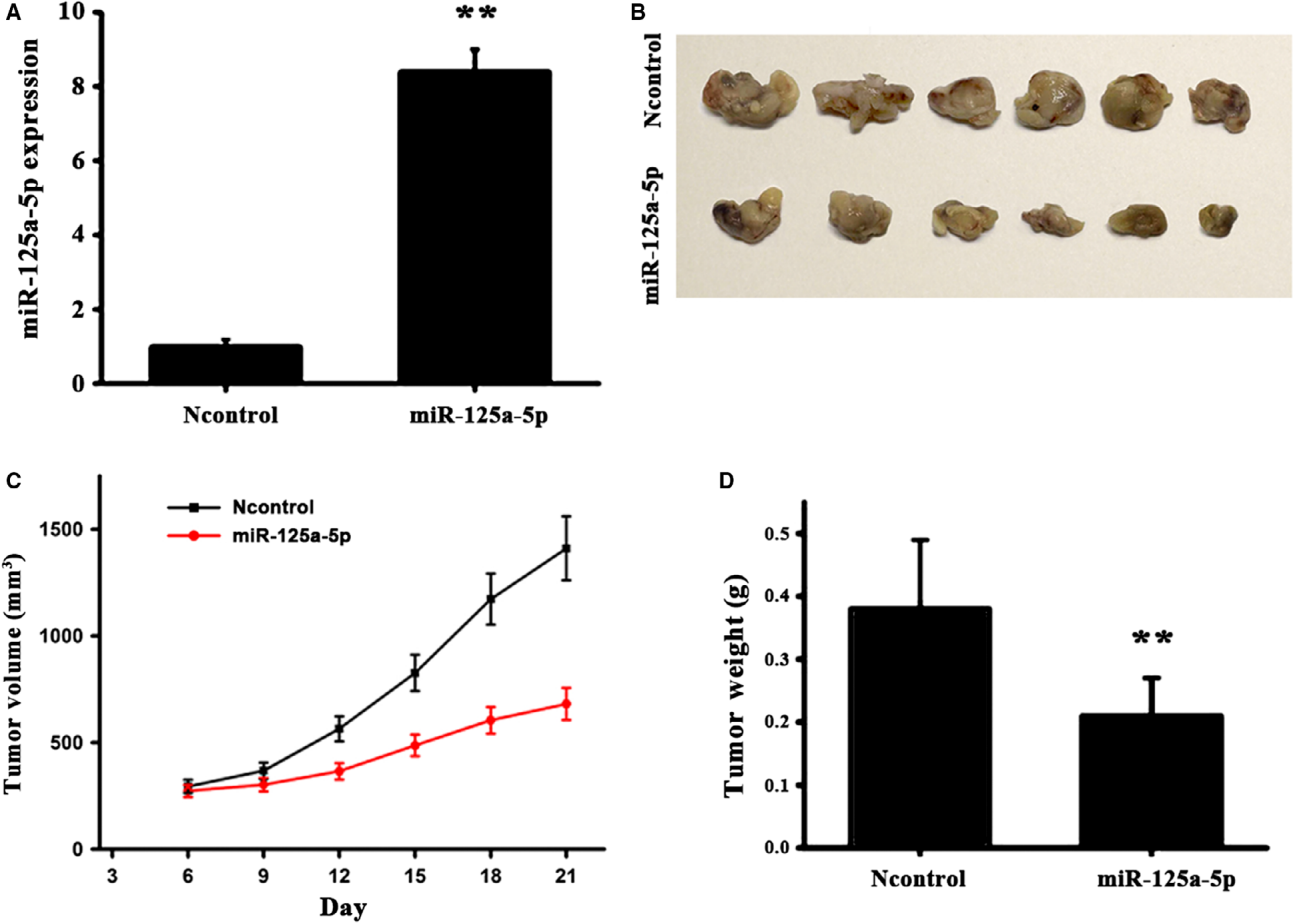
MiR‐125a inhibits tumor growth *in vivo*. (A) The expression level of miR‐125a in the mouse xenograft tumor mode was determined by reverse transcription‐quantitative polymerase chain reaction. (B) Tumor size of nude mice following subcutaneous injection with CT26 cells pretransfected with AgomiR‐125a or AgomiR‐NC. (C) Tumor volume growth curves. (D) Tumor weight. *N* = 6 per group. Scale bar = 1 cm. NC, negative control. Data are presented as mean ± SD. Student's *t*‐test. ^**^ *P* < 0.01.

**Figure 6:**
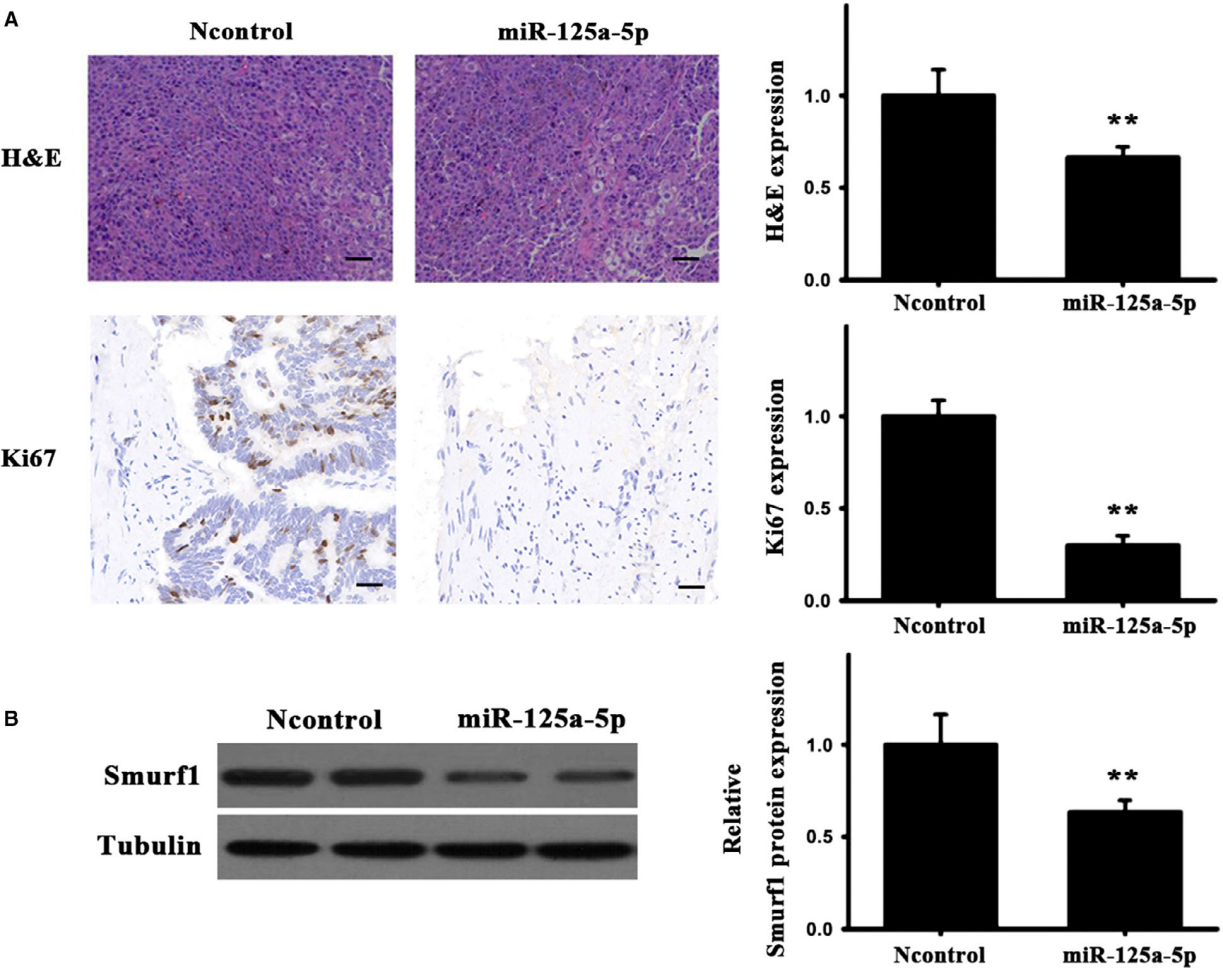
Effects of miR‐125a on Ki67 and Smurf1 expression. (A) Ki67 protein detection and quantification by H&E and immunohistochemical staining. (B) Smurf1 protein expression and quantification in tumors, as determined by western blotting. In the mouse xenograft tumor model, the expression of smruf1 protein was less than normal group. *N* = 3 per group. Three experiment replicates were performed. Scale bar = 50 μm. Data are presented as mean ± SD. Student's *t*‐test. ^**^ *P* < 0.01.

CT26 cells were used to establish a mouse xenograft tumor model, cells were transfected with miR‐125a agomir or the control, in order to elucidate the functional implications of miR‐125a *in vivo*. As presented in Fig. 5A–D, the treatment of miR‐125a strongly halted tumor growth (tumor size and weight), compared with the controls. Next, immunohistochemical staining was employed to examine the positive expression of Ki67 and Smurf1 proteins, with the results showing that the density of positive Ki67 and smurf‐positive cells was significantly lower in tumors treated with miR‐125a agomir than in those treated with the controls (Fig. 6A). Furthermore, western blot analysis indicated that the Smurf1 expression was lower in the observation group than that in the controls (Fig. 6B). These results confirmed that miR‐125a downregulates Smurf1, leading to a marked reduction in tumor growth.

## Discussion

MicroRNAs are critical regulators involved in numerous cellular processes, such as proliferation, migration, apoptosis, and differentiation. In addition, miRNAs have been closely associated with the initiation and progression of malignant tumors 25, 26. Although this study is not the first one to explore the implication of miR‐125a in cancer, the present study presented some novel findings. To begin with, Smurf1 was first shown here in to be a potential target of miR‐125a in CRC. Next, *in vitro* assays indicated that the overexpression of miR‐125a exerted antiproliferative and antimigratory effects in CT26 and SW620 cells. Finally, consistent with the *in vitro* results, it was identified that miR‐125a downregulated Smurf1 in a mouse tumor xenograft model. In combination, these results suggested that miR‐125a suppresses tumorigenesis in CRC by directly targeting Smurf1.

The abnormal expression of miRNAs has been demonstrated in a variety of human cancer types 27, 28, 29, 30, 31, 32. miR‐143 and miR‐145 have been shown to be downregulated in neoplastic colorectal tissue 33. Schepeler *et al.* 16 reported that the growth of three types of CRC cell lines (LS174T, DLD1, HCT116) was significantly inhibited when transfected with miR‐145, suggesting that miR‐145 may serve as a tumor inhibitor. miRNA‐143 could exert a critical effect in CRC cell proliferation by targeting the K‐ras gene, which is involved in various signaling pathways mediating cellular biological processes 34, 35. Recent studies have shown that miR‐125a functions as a tumor inhibitor of the malignant phenotype of CRC cells by binding to signal transducer and activator of transcription 3 36. In addition, miR–125a has been reported to exhibit an important function in inhibiting osteosarcoma cell migration and invasion by binding to matrix metalloproteinase‐11 (MMP‐11) 37. Furthermore, the miR–125a overexpression significantly inhibited the proliferation and metastasis of hepatocellular carcinoma by targeting MMP‐11 and vascular endothelial growth factor 38. Together, these studies suggested that miR‐125a plays a crucial role in the tumorigenesis of different types of cancer by targeting different genes. However, the association of miR‐125a with the pathogenesis and progression of CRC remains unclear. The present study was the first to confirm that miR‐125a markedly affected CT26, SW620 cell lines, and tumor tissue. The overexpression of miR‐125a significantly suppressed CT26, SW620 cell proliferation, and migration *in vitro* and inhibited tumor growth by downregulating Ki67 expression *in vivo*. However, the disadvantage of this study is that although miR‐125a has been reported to be downregulated in human colorectal cancer tissues, it has not been verified again in the paper. A follow‐up clinical study will be conducted to clarify the correlation between miR‐125a expression in human colon cancer and the prognosis of CRC treatment. In combination, these findings indicated that the modulation of cell proliferation and migration by miR‐125a contributed to the initiation and progression of CRC.

To further identify the underlying mechanisms of miR‐125a inhibiting cell growth, proliferation, and migration in CRC, it was demonstrated that Smurf1 is a potential target of miR‐125a. Smurf1 was the first strong oncogene candidate, since it has been known to be associated with the TGFβ signaling pathway. In general, TGFβ targets its receptors resulting in the phosphorylation of SMAD2/SMAD3, which then, in complex with SMAD4, regulates transcription. Hruban *et al.* 39 indicated that the TGFβ signaling pathway is normally disrupted via a mutation of SMAD4 and inactivation of TGFβR I and II in pancreatic cancer. Kwei *et al.* 40 reported Smurf1 as an amplified oncogene promoting cell invasion in pancreatic cancer, suggesting that Smurf1 may be a tractable drug target. Other studies have also revealed that the overexpression of Smurf1 in pluripotent mesenchymal cells and pre‐osteoblasts could repress osteoblast differentiation and reported that the Smurf‐transgenic mice presented with low bone mass due to reduced bone formation 41, 42. The present study was the first to reveal that miR‐125a can decrease the Smurf1 mRNA and protein expression levels in colon cancer cells by directly targeting the 3′‐UTR of Smurf1, which suggests that Smurf1 is a potent target of miR‐125a for CRC prevention and treatment.

## Conclusions

In conclusion, the present results demonstrated that the overexpression of miR‐125a can repress the malignant phenotype of CRC cells *in vitro* and inhibit tumor growth in xenograft mouse models *in vivo*, with Smurf1 as a potential and functional target. Collectively, the results of this study provide sufficient evidence that miR‐125a is a potential therapeutic target for CRC and that the miR‐125a/Smurf1 pathway may contribute to the development of new therapeutic strategies for CRC. Although this study is not the first one to explore the implication of miR‐125a in cancers, we provided the evidence that Smurf1 was one potential target of miR‐125a in CRC, which is some novel finding in this study.

## Conflict of interest

The authors declare no conflict of interest.

## Author contributions

DL, XX, and JC conceived and designed the project, DL and JM acquired the data, XX, JM, and JC analyzed and interpreted the data, and all authors wrote the paper.
